# Oropharynx pain, discomfort, and economic impact of transesophageal echocardiography for planned radio-frequency catheter ablation in patients with atrial fibrillation: A cross-sectional survey study

**DOI:** 10.1016/j.ijcha.2023.101266

**Published:** 2023-09-10

**Authors:** Rui Zeng, Xiaobo Pu, Shi Chen, Chunjia Chen, Yi Chen, Wendong Chen, Hua Fu

**Affiliations:** a West China Hospital of Sichuan University, Chengdu, China; b Changsha Normin Health Technology Ltd., Changsha, China; c Normin Health Consulting Ltd., Mississauga, Ontario, Canada; d Toronto Health Economics and Technology Assessment Collaborative, University of Toronto, Toronto, Ontario, Canada

**Keywords:** Atrial fibrillation, Transesophageal echocardiography, Pain, Discomfort, Satisfaction

## Abstract

**Background:**

To survey the unmet medical needs associated with atrium thrombus screening in Chinese patients with atrial fibrillation (AF) who underwent transesophageal echocardiography (TEE) for planned radio-frequency catheter ablation (RFCA).

**Methods:**

This cross-sectional survey study interviewed 300 patients who underwent their first TEE for planned RFCA. The surveyed information included patients' anxiety, oropharynx pain and discomfort, time expense, and patient satisfaction related to TEE examination. Patient preference for a new atrium thrombus screening technology, hospital length of stay (LOS) of RFCA, and hospital costs of RFCA in these surveyed patients were collected as well. Descriptive statistical methods were used to summarize the collected survey information.

**Results:**

Of the 300 interviewed patients, 36.3% reported anxiety before TEE examination, 58.6% reported oropharynx pain related to TEE, and 76.2% reported oropharynx discomforts, mainly including foreign body sensation (54.3%), dry heaves (33.8%), nausea (31.9%), and bleeding (22.9%). Even though 62.3% were satisfied with TEE, 84.3% preferred a new technology to replace TEE. Conducting outpatient TEE took more wait time (4.4 days vs. 0.1 days, p = 0.016) but led to significantly shorter hospital LOS (3.8 days vs. 6.4 days, p < 0.001) and significant lower hospital costs for RFCA (¥74,097 vs. ¥85,843, p < 0.001) than conducting inpatient TEE.

**Conclusions:**

Most AF patients experienced oropharynx pain and discomfort during or after TEE. Although more than half of AF patients were satisfied with TEE, most AF patients preferred a new technology to replace TEE for atrium thrombus screening. TEE was associated with economic impact on RFCA irrespective of TEE conducting settings.

## 1 Introduction

Transesophageal echocardiography (TEE) is a diagnostic imaging technique that uses high-frequency sound waves to create detailed images of the heart and its surrounding structures [1]. TEE has been used to screen the left atrial appendage, which is commonly associated with blood clots in patients with atrial fibrillation (AF) [2]. This information can be used to determine the best strategy for managing anticoagulation during and after the radiofrequency catheter ablation (RFCA) procedure. In addition, TEE can also be used to identify any structural abnormalities or defects in the heart, such as a patent foramen ovale or atrial septal defect, which may increase the risk of complications during RFCA [3].

TEE is conducted by using a specialized probe that is inserted through the mouth and down into the esophagus to obtain detailed images of the heart and its surrounding structures. Before TEE examination, the patient is typically asked to fast for several hours to empty the stomach and given a local anesthetic and a sedative to help with examination. After TEE examination is finished, the patient needs to be monitored for a short time to ensure that there are no complications. The patient may experience some throat discomfort or a sore throat after the examination. Even through TEE has been proven a safe examination, TEE is still challengeable in some patients due to anxiety, pain, discomfort, and underline comorbidities. Patients with AF are usually associated with old age and prevalent comorbidities (such as cardiovascular diseases, chronic obstructive pulmonary disease, and obesity), which could substantially increase the difficulties in performing the examinations similar to TEE (such as upper endoscopy) [4], [5], [6]. To improve the tolerance of TEE and facilitate TEE examination, general anesthesia is needed to perform TEE if the patient is unable to tolerate the examination under local anesthesia. Additionally, over half of patients reported poor experience with TEE due to stress, pain, and discomforts associated with the examination [7]. To our knowledge, there are no studies assessing patients’ experience and satisfaction with TEE evaluation that is required for planned RFCA in China. Hence, the present study aimed to explore the unmet needs of TEE examination and generate evidence to support the development of future management strategies addressing rising AF population alone with the aging population in China.

## 2 Materials and methods

This study was designed as a cross-sectional survey for AF patients who underwent TEE for planned RFCA in a tertiary care hospital (West China Hospital, Chengdu, China). The Ethical Review Committee of West China Hospital approved this study in March 2022 [Ethical approval number: 2022 Review (323)].

### 2.1 Patient selection

This survey study invited AF patients who underwent TEE examination for planned RFCA at West Hospital from March 2022 to November 2022. The study eligibility of the invited patients was assessed using the following inclusion and exclusion criteria. The inclusion criteria were: (1) patients aged 18–80 years old; (2) patients diagnosed with paroxysmal or persistent AF; (3) patients undergoing TEE examination for the first RFCA; (4) patients underwent TEE examination within one week before patient enrollment; (5) patients agreeing to participate this study by signing an informed consent form. The exclusion criteria were: (1) patients with a relapse from previous RFCA or a history of TEE assessment; (2) patients who had experienced cardiovascular events, including acute myocardial infarction, percutaneous coronary intervention, heart valve surgery, or coronary artery bypass surgery within 3 months before the study invitation; (3) patients underwent TEE for the management of a disease other than AF; (4) patients with insufficient communication capacity.

### 2.2 Survey package development

A survey package was developed to collect patient information (demographics: age, gender, weight, and height; socioeconomic status: educational level, employment status; clinical information: AF type, and comorbidities), patient setting (outpatient or inpatient), anxiety level before TEE [ measured by visional analogue scale (VAS), where 0 indicates no anxiety and 10 indicates severe anxiety], onsite management of uncontrolled blood pressure and/or heart rate before TEE, anesthesia mode, oropharynx pain level (measured by VAS, where 0 indicates no pain and 10 indicates severe pain) and types of oropharynx discomfort during and after TEE, overall satisfaction with TEE (measured by VAS, where 0 indicates extremely dissatisfied and 10 indicates very satisfaction; dissatisfaction was defined as VAS score as of 6 or less), and preference for new technology that can avoid the disadvantages of TEE. In addition, the survey gathered information on the time spent on booking a TEE appointment, wait time between TEE examination appointment booking and the date of undergoing the TEE examination, and the time involved in undergoing a TEE examination, which included the round-trip travel time from home to the hospital, waiting time after check-in, preparation, actual examination and observation time. The developed survey package was critically reviewed by interventional cardiologists conducting RFCA and the physicians conducting TEE examination in the hospital. In addition, the developed survey package was tested in a pilot of 10 patients who underwent TEE for planned RFCA if any unclear and confusing questions or formatting problems. The finalized survey package was converted to electronic version that allowed the research associate to obtain informed consents from patients and conduct the survey using electronic devices.

### 2.3 Statistical analysis

This study used descriptive statistical methods to summarize the survey information. Continuous survey data following symmetric distribution was summarized with mean and standard deviation and survey data, while median and interquartile range were used for continuous survey data with skewed distribution. Categorical survey data was summarized using percentages. To explore factors associated with patient reported outcomes (PRO), appropriate linear or logistic regression analyses methods were selected based on the distribution and nature of PRO. Simple linear regression was used for normally distributed PRO measured by VAS, and generalized linear regression was used for skewed VAS score of PRO. Multivariate logistic regression analyses were used for oropharynx discomfort (defined as reporting any types of oropharynx discomfort). Patients with pre-existing oropharynx pain or discomfort were excluded from the analyses for oropharynx pain and discomfort to avoid confounding effects.

The study used chi-squared test to compare the proportions of patients with preference for new technology to assess thrombus risk before planned RFCA in the surveyed patients stratified by patient characteristics, which included age (<or = 60 years vs. >60 years), gender (male vs. female), education level (high school below vs. high school or above), AF type (paroxysmal AF vs. persistent AF), comorbidity with cardiovascular disease (yes vs. no), and onsite management of uncontrolled blood pressure and heart rate (yes vs. no). The surveyed patients were followed up to collect hospital length of stay (LOS) and hospital costs associated with RFCA to compare the two outcomes between patients who underwent TEE examination as outpatients and inpatients using student *t* test (for hospital LOS) and Wilcoxon rank-sum test (for hospital costs). This study also conducted student *t* test to compare the wait time for TEE appointment and the time duration of TEE examination between outpatients and inpatients. Multivariate generalized linear regression analyses with adjustment of patient characteristics were conducted to confirm the difference in hospital LOS and hospital costs for RFCA between the patient settings of conducting TEE (outpatient vs. inpatient).

All statistical analyses were performed using R (4.2.2) software, and statistical significance was defined as a two-sided p-value <0.05.

## 3 Results

**Fig. 1:**
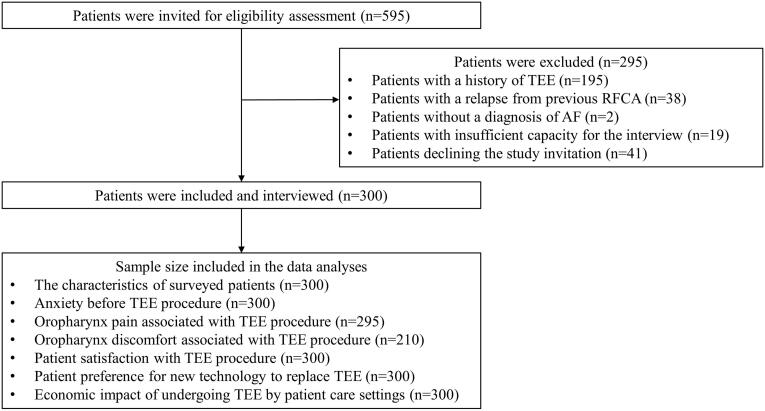
Patient enrollment flowchart and the sample size included in the data analyses.

During the observation period, 595 patients underwent TEE. Of these patients, 295 were excluded from the study due to meeting ineligibility criteria, including a history of TEE (233 patients), declining the study invitation (41 patients), inadequate communication capacity to conduct the survey (19 patients), or lacking a diagnosis of AF (2 patients). As a result, the study ultimately surveyed 300 patients with AF who had undergone TEE examination for planned RFCA at the study hospital. The patient enrollment flowchart is illustrated in Fig. 1.

### 3.1 The characteristics of the surveyed patients

The surveyed 300 patients included 94 outpatients and 206 inpatients admitted for planned RFCA. These patients had a mean age of 61.1 years (SD:10.3 years), a male proportion of 62.0%, and a mean BMI of 24.7 kg/m^2^ (SD: 3.3 kg/m^2^). Among these included patients, paroxysmal AF was the most common type, accounting for 69.7%. The main reported comorbidities were cardiovascular diseases (47.4%) and diabetes (13%). All patients underwent TEE examination under local anesthesia, and 13.7% required onsite management of uncontrolled blood pressure and heart rate onsite before TEE examination Table 1 summarizes the characteristics of the surveyed patients.

**Table 1:** Summary of the characteristics of the surveyed patients who underwent TEE examination for planned RFCA.

Patient characteristic	N	Mean/%	SD	Median	Q1	Q3	Min	Max
*Demographics*								
Mean age (years)	300	61.1	10.3	61.5	55.0	68.3	25.0	80.0
Male proportion	300	62.0%						
BMI	300	24.7	3.3	24.4	22.4	26.6	16.6	37.1

*Education level*								
Above high school	300	32.0%						

*Employment Status*								
Retired	300	52.0%						

*AF type*								
Persistent atrial fibrillation	300	30.3%						
Paroxysmal atrial fibrillation	300	69.7%						

*Comorbidity*								
Cardiovascular disease only	300	39.7%						
Diabetes only	300	5.3%						
Combination of diabetes and cardiovascular disease	300	7.7%						
Other comorbidities	300	1.0%						

*Anesthesia method*								
Local anesthesia	300	100.0%						
General anesthesia	300	0.0%						

*Onsite management of uncontrolled blood pressure and heart rate*								
Yes	300	13.7%						

TEE: transesophageal echocardiography, RFCA: radio-frequency catheter ablation, BMI: body mass index, AF: atrial fibrillation.

### 3.2 Anxiety before TEE examination

**Fig. 2:**
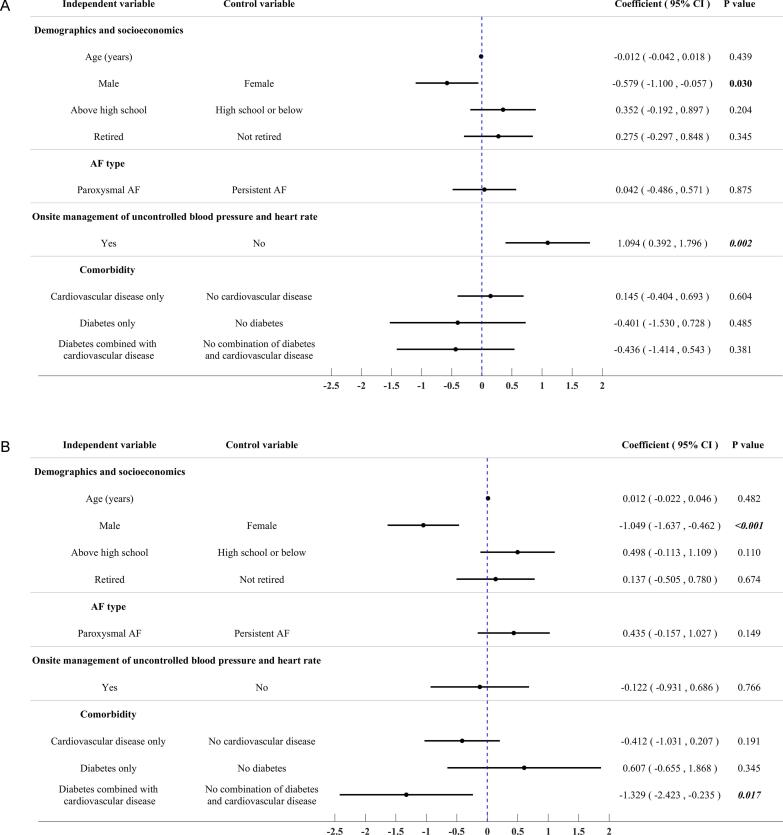

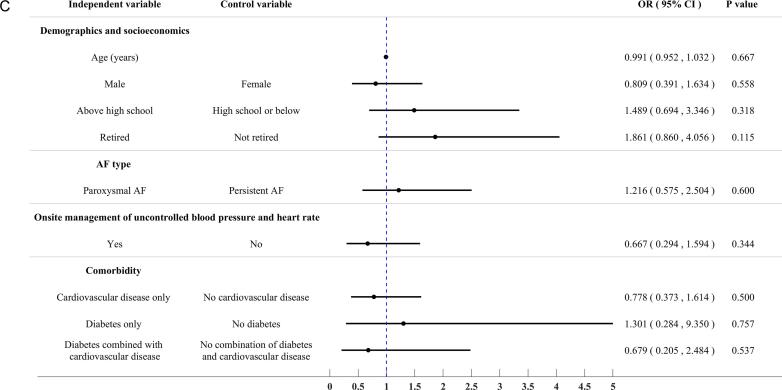
Results of multivariate regression analyses exploring predictors for the reported anxiety before TEE examination, oropharynx pain associated with TEE procedure, and oropharynx discomfort associated with TEE examination in the surveyed patients. (A) Anxiety before TEE examination (Multivariate linear regression analysis for anxiety VAS score); (B) Oropharynx pain associated with TEE examination (Multivariate linear regression analysis for oropharynx pain VAS score); (C) Oropharynx discomfort associated with TEE examination (Multivariate logistic regression analysis for the occurrence of any oropharynx discomforts).

Out of the 300 patients surveyed, 63.7% reported no anxiety, while 16.7% reported experiencing mild anxiety, defined as a score between 1 and 3 on the VAS. The remaining 19.7% of patients reported moderate to severe anxiety, defined as a score between 4 and 10 on the VAS. The average VAS score for anxiety in these surveyed patients was 1.4 (SD: 2.1). Multivariate linear regression analysis identified that male gender [coefficient −0.579, 95% confidence interval (CI) −1.100 to −0.057, p = 0.030] and management of uncontrolled blood pressure and heart rates (coefficient 1.094, 95% CI 0.392 to 1.796, p = 0.002) had significant associations with VSA score for anxiety [Fig. 2 (A)].

### 3.3 Oropharynx pain associated with TEE examination

Out of the 295 patients who did not report any oropharynx pain before TEE examination, 58.6% experienced oropharynx pain, which was defined as a visual analog scale (VAS) score greater than 0, during and/or after the examination. Of these 295 patients, 21.0% reported mild pain (VAS score from 1 to 3) and 37.6% reported moderate to severe pain (VAS score from 4 to 10). The mean VAS score for oropharynx pain during and/or after a TEE examination in these 295 patients was 2.5 (SD: 2.4). Multivariate linear regression analysis identified that male gender (coefficient −1.049, 95% CI −1.637 to −0.462, p < 0.001) and comorbidity of diabetes combined with cardiovascular disease (coefficient −1.329, 95% CI −2.423 to −0.235, p = 0.017) had significant associations with VAS score for oropharynx pain [Fig. 2 (B)].

### 3.4 Oropharynx discomfort associated with TEE examination

Of the initial 300 patients enrolled in the study, 90 were excluded due to pre-existing oropharynx discomfort, leaving 210 patients for analysis. Of these, 76.2% reported experiencing at least one discomfort during and/or after the TEE examination. The most reported discomforts were foreign body sensation (54.3%), dry heaving (33.8%), nausea and vomiting (31.9%), and bleeding (22.9%). A multivariate logistic regression analysis was performed to determine if there were any significant associations between patient characteristics and the risk of experiencing discomfort during and/or after the TEE examination. However, no significant associations were found [Fig. 2 (C)].

The reported anxiety before TEE examination, oropharynx pain associated with TEE examination, and oropharynx discomfort associated with TEE examination were summarized in Table 2.

**Table 2:** The reported anxiety before TEE examination, oropharynx pain associated with TEE examination, and oropharynx discomfort associated with TEE examination.

Patient reported outcome	N	Mean/%	SD	Median	Q1	Q3	Min	Max
*Anxiety before TEE examination*								
VAS score	300	1.447	2.142	0.000	0.000	3.000	0.000	10.000
No anxiety	300	63.7%						
Mild anxiety	300	16.7%						
Moderate to severe anxiety	300	19.7%						

*Oropharynx pain*								
VAS score	295	2.478	2.420	3.000	0.000	4.000	0.000	9.000
No pain	295	41.4%						
Mild pain	295	21.0%						
Moderate to severe pain	295	37.6%						

*Oropharynx discomfort*								
Any discomfort	210	76.2%						
Foreign body sensation	210	54.3%						
Retching	210	33.8%						
Nausea	210	31.9%						
Bleeding	210	22.9%						
Dysphagia	210	18.1%						
Difficulty speaking	210	15.7%						
Cough	210	10.0%						
Other discomfort	210	13.8%						

TEE: transesophageal echocardiography, VAS: visional analogue scale.

### 3.5 Patient satisfaction with TEE examination and preference for new technology

The average VAS score for overall satisfaction with TEE among the 300 surveyed patients was 6.7 (SD: 1.6). Of the surveyed patients, 37.7% were not satisfied with TEE, due to factors such as the time spent on the examination (34.7%), costs associated with TEE (51.4%), or oropharynx pain and discomfort (39.7%). Both multivariate linear regression analysis and multivariate logistic regression analysis did not reveal any significant associations between the patient characteristics collected and their overall satisfaction with TEE. However, male gender was associated with a strong likelihood to have a higher VAS score for overall satisfaction in multivariate linear regression analysis (coefficient 0.387, p = 0.054).

Among the patients surveyed, 84.3% expressed preference to undergo a new technology that would not cause pain or discomfort in the oropharynx for planned RFCA. Stratification analysis by patient characteristics showed that those with higher education level (above high school vs. high school or below) had a significantly higher proportion of preference for a new technology to replace TEE (90.6% vs. 81.4%, p = 0.040). Additionally, younger patients (60 years or below vs. above 60 years) had a higher proportion of preference for a new technology (88.0% vs. 81.0%, p = 0.095).

The reported PRO for satisfaction with TEE examination and preference for new technology from the surveyed patients are summarized in Table 3. The results of patient preference subgroup analyses are summarized in Table 4.

**Table 3:** The reported satisfaction with TEE examination and preference for a new technology to replace TEE examination from the surveyed patients.

Patient reported outcome	N	Mean/%	SD	Median	Q1	Q3	Min	Max
Reported satisfaction with TEE examination by perspective								
*All spent time related to TEE examination*								
*Satisfaction VAS score*	300	6.7	2.0	7.0	6.0	8.0	0.0	10.0
Dissatisfied: 0–6 points (%)	300	34.7%						
Satisfied: 7–10 points (%)	300	65.3%						

*All costs related to TEE examination*								
*Satisfaction VAS score*	72	5.8	2.0	6.0	5.0	7.0	0.0	9.0
Dissatisfied: 0–6 points (%)	72	51.4%						
Satisfied: 7–10 points (%)	72	48.6%						

*Pain and discomfort associated with TEE procedure*								
*Satisfaction VAS score*	300	6.6	1.9	7.0	5.0	8.0	0.0	10.0
Dissatisfied: 0–6 points (%)	300	39.7%						
Satisfied: 7–10 points (%)	300	60.3%						
*Overall*								
*Satisfaction VAS score*	300	6.7	1.6	7.0	6.0	8.0	0.0	10.0
Dissatisfied: 0–6 points (%)	300	37.7%						
Satisfied: 7–10 points (%)	300	62.3%						

Preference for a new technology to replace TEE examination								
Yes	300	84.3%						
No	300	1.0%						
Unsure	300	14.7%						

TEE: transesophageal echocardiography, VAS: visional analogue scale.

**Table 4:** Comparisons of the preference for new technology to replace TEE examination in the surveyed patients who were stratified by characteristics.

Age		60 years or blow			Above 60 years			P-value
		N	n	Mean/%	N	n	Mean/%	
Preference	Yes	142	125	88.0%	158	128	81.0%	0.095
	No	142	2	1.4%	158	1	0.6%	0.926
	Unsure	142	15	10.6%	158	29	18.4%	0.057

Education level		Above high school			High school or below			P-value

N	n	Mean/%	N	n	Mean/%

Preference	Yes	96	87	90.6%	204	166	81.4%	***0.040***
	No	96	0	0.0%	204	3	1.5%	0.554
	Unsure	96	9	9.4%	204	35	17.2%	0.076

Employment status		Retired			Not retired			P-value

N	n	Mean/%	N	n	Mean/%

Preference	Yes	156	133	85.3%	144	120	83.3%	0.647
	No	156	1	0.6%	144	2	1.4%	0.944
	Unsure	156	22	14.1%	144	22	15.3%	0.774

Comorbidity		Cardiovascular disease			No cardiovascular disease			P-value

N	n	Mean/%	N	n	Mean/%

Preference	Yes	144	124	86.1%	156	129	82.7%	0.416
	No	144	1	0.7%	156	2	1.3%	1.000
	Unsure	144	19	13.2%	156	25	16.0%	0.489

Onsite management of uncontrolled blood pressure and heart rate		Yes			No			P-value

N	n	Mean/%	N	n	Mean/%

Preference	Yes	41	38	92.7%	259	215	83.0%	0.113
	No	41	1	2.4%	259	2	0.8%	0.358
	Unsure	41	2	4.9%	259	42	16.2%	0.057

TEE: transesophageal echocardiography.

### 3.6 Economic impact of undergoing TEE

**Fig. 3:**
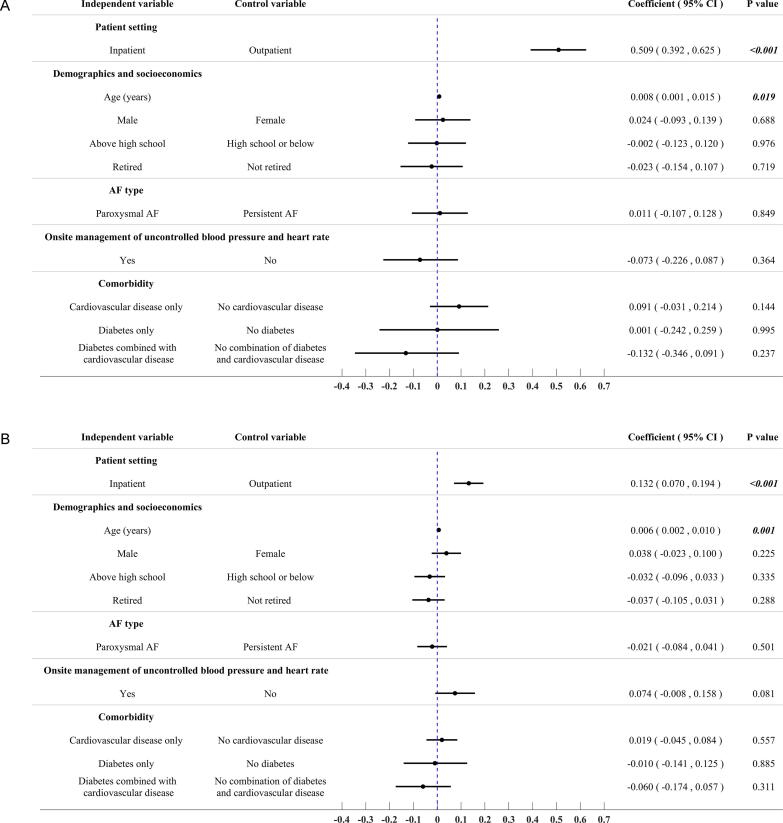
Results of multivariate regression analyses exploring predictors for hospital LOS and hospital costs associated with RFCA in the surveyed patients. (A) Hospital LOS of RFCA; (B) Hospital costs of RFCA.

Compared to surveyed inpatients, patients who underwent the TEE examination as outpatients had to schedule an appointment in advance, resulting in significantly longer waiting times (4.4 days vs. 0.1 days, p = 0.016) and longer onsite duration for TEE examination (4.6 h vs. 1.4 h, p < 0.001). However, outpatients who underwent the TEE examination experienced a significantly shorter hospital length of stay (LOS) (3.8 days vs. 6.4 days, p < 0.001) and significantly lower hospital costs for RFCA (¥74,097 vs. ¥85,843, p < 0.001) when compared to inpatients who were admitted to the hospital first before TEE examination. Multiple generalized linear regression analyses with adjustment of patient characteristics (demographics, socioeconomics, AF type, onsite management of uncontrolled blood pressure and heart rate, and comorbidities) confirmed the statistical significance for shorter hospital LOS (coefficient 0.509, 95% CI 0.392 to 0.625, p < 0.001) and hospital costs (coefficient 0.132, 95% CI 0.070 to 0.194, p < 0.001) associated with RFCA in the patients who underwent TEE examination as inpatients. The results for the comparisons of hospital LOS and hospital costs for RFCA between patient settings are summarized in Table 5. The results of multivariate generalized linear regression analyses exploring the predictors for the two outcomes are illustrated in Fig. 3.

**Table 5:** Comparisons of spent time related to TEE examination, hospital LOS of RFCA, and hospital costs of RFCA in the surveyed patients stratified by patient settings.

Patient setting	Inpatient									Outpatient									P-value
	N	n	Mean/%	SD	Median	Q1	Q3	Min	Max	N	n	Mean/%	SD	Median	Q1	Q3	Min	Max	
Wait time for TEE appointment (days)	206	2	0.1	0.1	0.1	0.1	0.1	0.0	0.2	94	91	4.4	8.9	2.0	1.0	3.5	0.0	60.0	***0.016***
Duration of TEE examination (hours)	206	200	1.4	0.7	1.0	1.0	2.0	0.5	4.0	94	89	4.6	4.8	4.0	2.0	5.0	1.0	30.0	***<0.001***
Hospital LOS of RFCA (days)	206	206	6.4	3.4	6.0	5.0	7.0	3.0	25.0	94	94	3.8	1.5	3.0	3.0	4.0	2.0	11.0	***<0.001***
Hospital costs of RFCA	206	206	¥85,843	¥26,709	¥77,517	¥73,995	¥86,718	¥34,830	¥230,412	94	94	¥74,097	¥6,958	¥72,694	¥70,637	¥75,969	¥59,751	¥109,930	***<0.001***

TEE: transesophageal echocardiography, RFCA: radio-frequency catheter ablation, LOS: length of stay.

## 4 Discussion

This cross-sectional survey was conducted at the largest AF treatment center in Southwest China, where RFCA is commonly used to treat AF. TEE evaluation is critical for RFCA as it helps to identify the cause of arrhythmia, evaluate heart function, identify the risk of complications, and assess the feasibility of the planned procedure. Despite TEE being generally considered safe and rarely causing serious complications, this survey study suggests that the pre-examination anxiety, oropharyngeal pain and discomfort, and undergoing additional management to control their blood pressure and heart rate could cause unpleasant experience, which could make overall satisfaction with TEE in the surveyed patients was much lower than that for other similar examinations, such as conventional upper endoscopy (62.3% vs. 86.6%) [8]. Thus, most surveyed patients preferred a new technology that could achieve the same goals as TEE but without causing the unpleasant experiences of TEE examination.

Similar to upper endoscopy, TEE is conducted by inserting endoscope through esophageal. However, TEE requires manipulation of the endoscope for proper positioning, which can cause more minor oropharyngeal and esophageal injury (0.1% to 13%) [9]. Patients with AF who take anticoagulants to prevent stroke are more likely to experience bleeding during TEE. The reported bleeding associated with TEE in this study was 22.9%, while most studies report a rate of <1% in patients who undergo upper gastroendoscopy [10]. In addition, the other characteristics of AF patients could further worsen the experience with TEE. For example, AF patients usually had old age with highly prevalent cardiovascular diseases that could cause anxiety and increase blood pressure and heart rate. Consequently, a certain proportion of patients, 13.7% in our study, require additional onsite management of uncontrolled blood pressure and heart rates. While all surveyed patients underwent TEE under local anesthesia, a previous study reported that about 2% of patients might not tolerate the insertion of the TEE probe and may require general anesthesia to conduct TEE [11]. This study also explored patient characteristics associated with anxiety, oropharynx pain, and oropharynx discomfort. Like previous studies [12], [13], [14], male patients in this study had better tolerance with TEE examination by experiencing lower levels of anxiety and oropharynx pain than female patients. Interestingly, patients with combined comorbidities of cardiovascular diseases and diabetes were found to be less sensitive to oropharynx pain than those without comorbidities. This reduced sensitivity to pain in diabetes patients may result from neuropathy, a common complication of diabetes [15], which can lead to injuries or infections going unnoticed. Therefore, more attention and cautions are necessary when conducting TEE in older patients with diabetes. The reported oropharynx pain and discomfort from the surveyed patients in this study are well aligned with previous research. A French study surveyed 1,718 patients who underwent TEE for stroke risk assessment [7]. In this French study, 62.4% of the surveyed patients reported an unpleasant experience with the TEE examination, mainly due to nausea (38.6%), pain (24.4%), and breathing difficulties (16.6%). Additionally, these patient-reported outcomes in this study are highly consistent with the reported complications associated with TEE examination for RFCA and other structural cardiac interventions in clinical studies. According to published observational studies [16], [17], [18], 30 to 40% of patients could develop endoscopically confirmed esophageal lesions and dysphagia after TEE examination. Moreover, the risk of developing esophageal lesions after TEE examinations could increase by 4 times in patients with abnormal esophagogastroduodenoscopy [18]. When TEE was used for structural cardiac interventions, the risk of complications associated with TEE examination was significantly related to TEE examination time [18], [19]. For each 10-minute increment in TEE examination time, the risk of developing complex esophageal lesions increased by about 30% [18]. Thus, the clinical eligibility of TEE examination still needs careful evaluation to control the complications of TEE and improve the patient experience with TEE examination. The collected economic information related to TEE examination in both inpatient and outpatient settings allowed a supplementary analysis to further explore the economic impact of TEE examination in Chinese AF patients who underwent RFCA. This supplementary analysis suggested that TEE examination could be associated with substantial time costs irrespective of patient setting. This study found that undergoing TEE in the inpatient setting was likely to aggravate pressure for the shortage of hospital beds, extend hospital LOS and subsequently increase hospital costs. To reduce this pressure on the efficiency of hospital beds, patients are often asked to undergo TEE before the hospital admission for RFCA. However, conducting TEE in outpatient setting could substantially increase patient waiting time and delay RFCA. The economic impact of TEE examination is unlikely to help with addressing the growing the medical needs of patients with AF in China.

Based on the reported disadvantages of TEE examination from the surveyed patients, it is not surprising that dissatisfaction with TEE examination in the surveyed patients (37.7%) was almost tripled when compared to other similar examinations (such as upper endoscopy) (13.4%) [8]. This might explain why over 80% of patients indicated a preference for a new technology that can achieve the same goal as TEE but avoid its reported disadvantages. In addition, patients who had higher levels of education and were younger in age were more likely to prefer new technology. While the motivations behind patients' preference for new technology were not explored in this study, it is understandable that older AF patients with comorbidities may have poorer tolerance for TEE than those younger patients who usually have fewer comorbidities. Despite all surveyed patients in this study undergoing TEE examination under local anesthesia, previous studies have reported that about 2% of patients may require general anesthesia due to a lack of tolerance for TEE examination under local anesthesia [11]. Conducting TEE examination under general anesthesia further increases health resource utilization and medical costs of AF management through RFCA. Thus, the poor experience and dissatisfaction associated with the TEE examination could discourage the use of RFCA and impede the effort to control the rapidly rising disease burden of AF in China.

To address these issues discussed above, alternatives to TEE have been developed to improve the performance of RFCA. For example, intracardiac echocardiography (ICE) has been increasingly used for RFCA as it provides higher resolution images of the heart and its structures than TEE [20] and improve thrombus detection in the left atrial appendage [21]. By directly inserting a probe into the heart, ICE can be conducted with RFCA, providing real-time visualization of cardiac structures and guiding the placement of ablation catheter during RFCA procedure. This eliminates oropharynx pain and discomfort and improves the chance for patients to receive RFCA. Further, conducting ICE with RFCA could improve turnover rate of hospital bed without delaying RFCA and extending hospital LOS of RFCA as TEE. With the superior capacity to detect thrombus in the left atrial appendages and the additional benefits listed above, ICE has become a desirable alternative for TEE and highly recommended for the AF patients who are unlikely to tolerate TEE due to older age and comorbidities [22].

The survey study has some limitations that should be considered when interpreting the findings. One limitation is that the study did not collect information on the experience, satisfaction, and preference for new technology to replace TEE examination from healthcare providers who are involved with RFCA procedures for AF patients. This limits the study's ability to fully demonstrate the challenges and disadvantages of TEE examination. Additionally, the study only surveyed patients from a single RFCA treatment center, which may limit the generalizability of the findings to AF patients across China who have different characteristics, cultures, and socioeconomic status. Furthermore, the study did not investigate the potential reasons for extended hospital LOS and increased hospital costs of RFCA for AF patients who underwent TEE examination after hospital admission for RFCA. Finally, the study did not elicit patient willingness-to-pay for the new technology that can replace TEE examination, which could overestimate patient preference for the new technology if its cost is not considered. These limitations should be addressed in future research.

In conclusion, this comprehensive survey study showed that TEE examination was associated with high incidences of anxiety before the examination, oropharynx pain, and discomfort, which could reduce patient satisfaction with TEE examination and increase their preference for a new technology to replace TEE examination for planned RFCA. The negative effects of TEE examination, such as delaying RFCA and increasing hospital LOS and costs, further support the need for alternative technologies to meet the demands of RFCA for AF in China.

**Funding**

This study was funded by Johnson and Johnson Medical (Shanghai) Ltd.

## CRediT authorship contribution statement

**Rui Zeng:** Conceptualization, Methodology, Formal analysis, Investigation, Data curation, Project administration, Writing – original draft. **Xiaobo Pu:** Conceptualization, Methodology, Supervision, Writing – original draft. **Shi Chen:** Methodology, Formal analysis, Investigation, Data curation, Writing – review & editing. **Chunjia Chen:** Formal analysis, Investigation, Writing – review & editing. **Yi Chen:** Formal analysis, Investigation, Supervision, Writing – review & editing. **Wendong Chen:** Methodology, Data curation, Supervision, Writing – original draft. **Hua Fu:** Conceptualization, Methodology, Data curation, Supervision, Writing – original draft, Project administration, Funding acquisition.

## Declaration of Competing Interest

The authors declare the following financial interests/personal relationships which may be considered as potential competing interests: Chunjia Chen, Yi Chen, and Wendong Chen are employed by contract research organizations that receive industry funds to conduct health economics and outcomes research. Other authors declare that the research was conducted in the absence of any commercial or financial relationships that could be construed as a potential conflict of interest.
